# Disparity in Retention in Care and Viral Suppression for Black Caribbean-Born Immigrants Living with HIV in Florida

**DOI:** 10.3390/ijerph14030285

**Published:** 2017-03-09

**Authors:** Elena Cyrus, Christyl Dawson, Kristopher P. Fennie, Diana M. Sheehan, Daniel E. Mauck, Mariana Sanchez, Lorene M. Maddox, Mary Jo Trepka

**Affiliations:** 1 Center for Substance Use and HIV/AIDS Research on Latinos in the United States (C-SALUD), Florida International University, 11200 SW 8th St, Miami, FL 33199, USA; cdawson@fiu.edu (C.D.); msanche@fiu.edu (M.S.); 2 Department of Epidemiology, Robert Stempel College of Public Health and Social Work, Florida International University, 11200 SW 8th St, Miami, FL 33199, USA; kfennie@fiu.edu (K.P.F.); dsheehan@fiu.edu (D.M.S.); dmauck@fiu.edu (D.E.M.); trepkam@fiu.edu (M.J.T.); 3 HIV/AIDS Section, Florida Department of Health, 4052 Bald Cypress Way, Tallahassee, FL 32399, USA; lorene.maddox@flhealth.gov

**Keywords:** Caribbean, immigrant, human immunodeficiency virus, retention, suppression, disparity

## Abstract

(1) The study aim was to assess disparities in non-retention in HIV care and non-viral suppression among non-Hispanic Black Caribbean immigrants living with HIV in Florida. (2) We analyzed cases involving individuals, aged ≥13, who met CDC HIV case definition during 2000–2014. Chi square test was used to evaluate differences in non-retention and non-viral suppression by country of origin/race/ethnicity. Multilevel logistic regressions with three referent groups [US-born Blacks, Hispanics, and non-Hispanic Whites (NHWs)] were used to estimate adjusted odds ratios (aOR). (3) Caribbean-born Blacks were less likely to be retained in care or be virally suppressed than US-born Blacks, Hispanics, and NHWs. Bahamians, Haitians, and Trinidadians and Tobagonians had increased odds of non-retention (aOR 3.13, 95% confidence interval [CI] 2.40 –4.10; aOR 1.52, 95% CI 1.40–1.66; aOR 2.30, 95% CI 1.38–3.83), and non-viral suppression (aOR 3.23, 95% CI 2.48–4.21; aOR 1.82, 95% CI 1.68–1.98; aOR 1.76, 95% CI 1.06–2.90) compared with NHWs. (4) Caribbean-born Blacks living with HIV infection are less likely than other racial/ethnic groups to be retained in care and/or achieve viral suppression. Further research is urgently needed to determine social, cultural, and biological factors that contribute to this disparity.

## 1. Introduction

The rate of HIV diagnoses in Florida is almost three times the national average [1]. Reduction in HIV incidence is possible if a larger proportion of persons living with HIV are consistently engaged and retained in care [2]. Retention in care is a critical factor for persons living with HIV to achieve viral suppression. Persons living with HIV who are in routine care and achieve undetectable viral loads minimize the chances of transmitting the virus to HIV-uninfected individuals [3].

Just over one percent of the US population is composed of Caribbean immigrants [4]. Florida is home to approximately 40.0% of the total Caribbean-born population in the United States [5]. From 2001 to 2007, Caribbean-born immigrants, primarily from Haiti, Jamaica, Trinidad and Tobago, and the Bahamas, comprised approximately 54.1% of the cases of HIV foreign-born Blacks in the United States [6]. Haitian-born immigrants continue to be at highest risk for HIV acquisition, accounting for 16% of all HIV cases in South Florida (Miami-Dade, Broward, and Palm Beach Counties) while representing only 2% of the total Florida population [7].

Compared to other populations in Florida, Caribbean immigrants are more likely to be screened later for HIV, resulting in delayed diagnosis [8]. Delayed diagnosis in Caribbean populations suggest other gaps in the HIV care continuum, including insufficient access to and/or engagement in care once diagnosed, leading to impaired disease management and lack of viral suppression, which can also impact primary prevention of HIV in uninfected populations [9].

Factors impacting the HIV continuum of care of Black Caribbean immigrants are under-researched. The main aim of the present study is to partially address this gap by assessing the disparity of non-retention in HIV care and non-viral suppression for non-Hispanic Black Caribbean-born immigrants.

## 2. Materials and Methods

### 2.1. Study Sample

We used de-identified records, obtained from the Florida Department of Health (DOH) Enhanced HIV/AIDS Reporting System (eHARS), of Florida residents aged 13 years or older who were diagnosed with HIV infection from 2000 to 2014 and met the Centers for Disease Control and Prevention (CDC) case definition for HIV [10]. Data in eHARS are sourced primarily from health care provider reports, laboratory reports, and data extracted from medical records by county health department staff.

Retention in care during 2015 was defined as engagement in care two or more times at least three months apart during 2015. We used an expanded definition for retention in care that took advantage of ADAP and Ryan White data to ensure that all medical encounters were captured. The standard definition of retention in care uses only evidence of a laboratory test [11]; however, given the additional information we have linked to our Florida surveillance data, we chose to use a more conservative measure. A person was engaged in care if there was evidence of at least one documented laboratory test, a prescription filled through the AIDS Drug Assistance Program (ADAP) (for those in ADAP), or a physician visit documented in one of the Ryan White databases (for those receiving services through the Ryan White HIV/AIDS program). HIV viral load suppression during 2015 was defined as having a viral load of <200 copies/mL in the last laboratory test performed during 2015. The last viral load test during the measurement year is used by the US Department of Health and Human Services HIV/AIDS Bureau to measure program performance [12].

Non-Hispanic Whites (NHW), US-born Blacks, Hispanics, and all foreign-born Blacks from non-Spanish speaking Caribbean countries were included in the analysis to assess disparities affecting Caribbean-born Blacks. Non-Hispanic White individuals living with HIV were used as a referent group because we hypothesized that this group had the greatest contrast with Caribbean–born persons living with HIV, and would therefore highlight disparities affecting Caribbean-born Blacks. Hispanic immigrants, who may have also been from the Caribbean region [13], were used to explore ethnic differences and or/similarities. US-born Blacks were selected as the final referent group to eliminate race as a confounder between Caribbean- and US-born Blacks, allowing for other contextual variables, such as country of origin or culture, to be considered as explanatory variables for the two primary outcomes.

Caribbean countries with fewer than 70 HIV cases were grouped together for analysis [Guyana (n = 26), Turks and Caicos (n = 20), Barbados (n = 18), St. Lucia (n = 14), Dominica (n = 10), Antigua and Barbuda (n = 6), Grenada (n = 8), St. Vincent and the Grenadines (n = 7), Bermuda (n = 2), British Virgin Islands (n = 5), St. Kitts and Nevis (n = 4), and Cayman Islands (n = 2)]. Countries that were Departments or Constituents of other countries (Dutch Antilles and French Guiana), were excluded. Records were excluded from the analysis if: HIV diagnosis occurred under 13 years of age (n = 270), missing month and year of HIV diagnosis (n = 79), missing country of birth (n = 907), missing or invalid residential ZIP code (n = 1306), or HIV diagnosis occurred at a correctional facility (n = 3195).

### 2.2. Individual-Level Characteristics

Individual-level variables available in the eHARS dataset for cases included current residential ZIP code; month and year of HIV diagnosis and AIDS diagnosis (if applicable); country of birth; age at HIV diagnosis; sex; race/ethnicity; HIV transmission mode; and whether the diagnosis occurred at a correctional facility. AIDS case definition was met if the person’s medical record indicated the development of an AIDS-defining illness, a CD4 lymphocyte count <200 cells/μL, or CD4% of total lymphocytes <14 [14]. People were classified as being born in the United States if they were born in mainland United States, Hawaii, Alaska, Guam, or the US Virgin Islands. Individuals from the US Virgin Islands were included with mainland US instead the Caribbean because of the similarity in terms of access to care. Puerto Ricans were included among the Hispanic group. Individuals with a reported mode of transmission of men who have sex with men (MSM) combined with injection drug use (IDU) were grouped with those who reported mode of transmission as IDU only, because, of the two risk categories, IDU is generally higher risk than MSM.

### 2.3. Neighborhood-Level Characteristics

Following a previously used procedure to create a socio-economic status (SES) index (described elsewhere with 2002–2008 American Community Survey estimates [15]), we used 2009–2013 American Community Survey estimates in the present study to create the SES index for our analysis. To categorize current ZIP code tabulation areas (ZCTA) into rural or urban, we used Categorization C of Version 2.0 (University of Washington, Washington, WA, USA) of the Rural-Urban Commuting Area (RUCA) codes, developed by the University of Washington WWAMI Rural Research Center [16].

### 2.4. Analytic Plan

Individual- and neighborhood-level data were merged by matching the current ZIP code with the ZIP code’s corresponding ZCTA. First, we compared individual- and neighborhood-level characteristics by country of birth and ethnicity. We used the Cochran-Mantel-Haenszel general association statistic for individual-level variables controlling for ZCTA, and the chi-square test for neighborhood-level variables. Multi-level (Level 1: individual; Level 2: neighborhood) logistic regression modeling was used to account for correlation among cases living in the same neighborhood. To explore varying disparity, three referent groups were used: US Blacks, Hispanics, and non-Hispanic Whites. Crude and adjusted odds ratios and 95% confidence intervals were calculated comparing cases by country of birth and ethnicity. To identify unique predictors of viral suppression and retention for Caribbean-born Blacks, separate models were estimated stratifying by country of birth. For the stratification, results are only presented for Bahamas, Haiti, and Jamaica—there were no statistically significant results for other Caribbean countries, and the model did not converge for Trinidad and Tobago due to a smaller sample size. Odds ratios were adjusted for year of HIV diagnosis, sex at birth, age, mode of HIV transmission, AIDS diagnosis by 2015, neighborhood SES, and rural/urban status. SAS software, version 9.4 (SAS Institute, Cary, NC, USA) was used to conduct analyses [17]. The study was reviewed and approved by a local Institutional Review Board, and the Florida Department of Health designated this study as non-human subjects research.

## 3. Results

Demographic characteristics of the study population are presented in Table 1. Haitians and Bahamians had a more even gender distribution among Caribbean countries, while Trinidadians and Tobagonians, Jamaicans, and US-born Blacks had a higher percentage of males living with HIV; Hispanics and NHWs also had the highest percentages of male cases. US-born Blacks, Bahamians, and Hispanics had the most individuals diagnosed in the 13–24 year-old age group (23.2%, 15.9%, and 12.8%), while individuals from Haiti, Jamaica, and Trinidad and Tobago had the most cases 50 years of age and older (24.7%, 21.8%, and 20.0%). Compared to Hispanics and NHWs, the Caribbean groups and US-born Blacks had a lower proportion of people reporting MSM as mode of HIV transmission. Compared to other racial/ethnic groups, NHW cases (25.4%), cases from Trinidad and Tobago (38.6%), and Hispanic cases (42.7%) had a lower proportion of people who had an AIDS defining illness by 2015. More Jamaicans and ‘Other’ Caribbean immigrants reported living in a ZCTA in the highest SES quartile compared to other Caribbean countries (10.4%, 9.4%); and NHWs and Hispanics had a higher percentage of individuals in the two highest SES ZCTA quartiles compared to US-born Blacks (21.6%, 12.9%).

**Figure 1:**
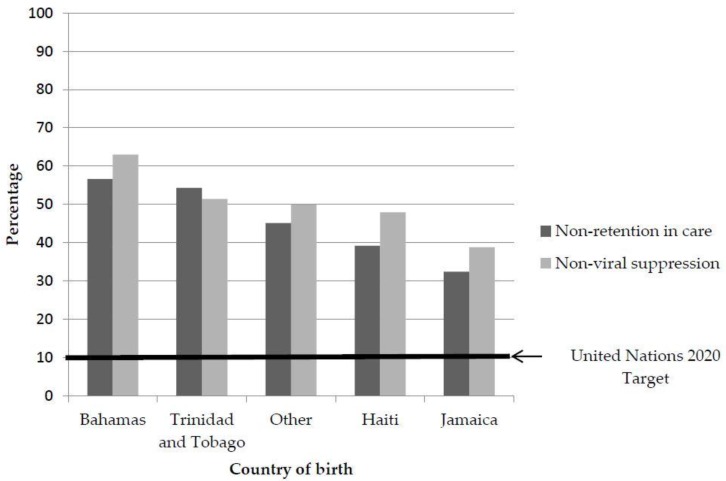
Non-retention and non-viral suppression by country of birth for persons living with HIV, 2015.

The analysis demonstrated that none of the groups is close to attaining the 2020 United Nations goal of 10% or less of HIV infected individuals being non-retained in care or achieving viral suppression [18], and non-Hispanic Caribbean immigrants are far from this goal (Figure 1). Overall, 33.2% of the population was not retained in care, and, 39.3% did not achieve viral suppression (data not presented). Of all the groups in the present analysis, persons living with HIV from Bahamas, Trinidad and Tobago, and Haiti had the highest percentages of non-retention and non-viral suppression (non-retention: 56.6%, 54.3%, 39.2%; non-viral suppression: 63.0%, 51.4%, 47.9%).

### Multivariate Analysis

#### Non-Retention in Care

HIV cases from Jamaica did not differ statistically from any of the reference groups, and Other Caribbean individuals were not different compared to US-born Blacks in non-retention in care (Table 2). For the remaining Caribbean countries, disparities were greater when compared to NHWs, followed by Hispanics, and US-born Blacks. In the fully adjusted models, the rates of non-retention were highest for Bahamas, Trinidad and Tobago, and Haiti, and the disparity was greatest compared to non-Hispanic Whites (retention adjusted odds ratio [aOR] 3.13, 95% confidence interval [CI] 2.40–4.10; viral suppression aOR 2.30, 95% CI 1.38–3.83; 1.52, 95% CI 1.40–1.66).

The post-hoc stratification revealed some insight into contributing variables to the results (Table 3). Other or unknown mode of HIV transmission was associated with increased odds of non-retention for all Caribbean countries (Bahamas aOR 4.62, 95% CI 1.78–12.18; Haiti aOR 3.46, 95% CI 2.82–3.72; Jamaica OR 2.18, 95% CI 1.28–3.71.); however, not having AIDS by 2015 was a risk factor for non-retention (aOR 2.80, 95% CI 1.49–5.25; Haiti aOR 3.24, 95% CI 2.82–3.72; Jamaica aOR 2.84, 95% CI 1.95–4.12). For Bahamians, individuals who were diagnosed between 2000–2004 were more likely not to be retained in care (aOR 3.02, 95% CI 1.42–6.44). For Haitian and Jamaican HIV-infected individuals, there were greater odds of non-retention if they were diagnosed in earlier years (Haiti: 2000–2004 aOR 1.90, 95% CI 1.60–2.25; 2005–2009 aOR 1.44, 95% CI 1.20–1.72. Jamaica: 2000–2004 aOR 1.67, 95% CI 1.05–2.65; 2005–2009 aOR 1.60, 95% CI 1.04–2.46), and if they were male (Haiti: 1.46, 95% CI 1.27–1.68. Jamaica: aOR 2.28, 95% CI 1.50–3.48). Additional protective factors for Haitians and Jamaicans were being diagnosed at an older age, and for Haitians only, living in rural areas (aOR 0.21, 95% CI 0.05–0.85).

#### Non-Viral Suppression

For this clinical outcome, individuals from Bahamas and Haiti consistently presented as being less likely to achieve viral suppression compared to all three referent groups (Table 4). Similar to the non-retention results, the greatest disparity occurred in the comparison to NHWs (aOR 3.23, 95% CI 2.48–4.21; aOR 1.82, 95% CI 1.68–1.98). Jamaicans and Trinidadians and Tobagonians only had statistical differences compared to NHWs, but not to any other racial/ethnic group (aOR 1.29, 95% CI 1.09–1.52; aOR 1.76, 95% CI 1.06–2.90). The one exception was that Jamaicans were more likely to achieve viral suppression compared to US-born Blacks (aOR 0.83, 95% CI 0.70–0.97).

The post-hoc stratification for non-viral suppression (Table 5) revealed higher odds for Bahamians, Haitians and Jamaicans diagnosed in earlier years (Bahamas: 2000–2004 aOR 2.26, 95% CI 1.07–4.75; Haiti: 2000–2004 aOR 1.65, 95% CI 1.40–1.93; 2005–2009 aOR 1.42, 95% CI 1.20–1.67. Jamaica: 2000–2004 aOR 1.89, 95% CI 1.26–2.84), or if the individual was a Haitian or Jamaican male (aOR 1.33, 95% CI 1.16–1.52, aOR 2.37, 95% CI 1.60–3.52). Other or unknown mode of HIV transmission was associated with increased non-viral suppression for individuals from Bahamas and Haiti (aOR 7.42, 95% CI 2.25–22.44; aOR 2.51 95% CI 2.07–3.06). Being diagnosed at an older age was protective for Jamaicans (25–49 years of age aOR 0.46, 95% CI 0.27–0.78; ≥ 50 years of age aOR 0.29, 95% CI 0.16–0.56); and it should be noted that Haitians approached significance for the same trend in age at diagnosis. Not having an AIDS diagnosis by 2015 was associated with increased odds of non-viral suppression for all three countries (Bahamas aOR 2.59, 95% CI 1.37–4.89; Haiti aOR 2.17, 95% CI 1.91–2.48; Jamaica aOR 1.96, 95% CI 1.39–2.77). For Haitians only, being MSM or residing in a rural area was protective and lowered the likelihood of non-suppression (aOR 0.76, 95% CI 0.61–0.96; aOR 0.21 95% CI 0.06–0.78).

## 4. Discussion

The proportion retained in care or achieving viral suppression was lowest for those in the Bahamas, Haiti, and Trinidad and Tobago. These countries also account for the highest HIV rates in the Caribbean region [18,19]. Bahamians’ odds were least favorable for both outcomes, and Trinidad and Tobago and Haiti were second for non-retention and non-viral suppression, respectively compared to NHWs.

As hypothesized, the disparity was greatest between the Caribbean groups and non-Hispanic Whites. Caribbean individuals also did worse than Hispanics for both outcomes. Different immigration policies between the two dominant Caribbean and Hispanic countries in the population may be part of the explanation for the differences between Hispanics and non-Hispanic Caribbean-born Blacks. Compared to US-born Blacks, persons living with HIV from Caribbean countries were also less likely to be retained in care or achieve undetectable viral loads, implying that the disparity may not fully be accounted for by race, but that other cultural and socio-economic considerations should be considered.

Hispanics living with HIV in South Florida are predominantly from the Caribbean region, specifically Cuba [20,21]. Among Black persons living with HIV in Florida, the majority are from Haiti [4,22]. During the time of the study, U.S. immigration policies for Cuba and Haiti differed in that Cuban immigrants were provided a level of state protection that facilitated easier access to medical care and other social services [21]. Therefore, despite both immigrant groups having strong ethnic ties with substantial receiving communities in South Florida [23,24], the differing socio-political context may increase HIV risk for Haitians over Cubans, and decrease Haitian individuals living with HIV subsequent engagement and retention in care, and disease management. Although immigration policy may not have a direct impact on individuals in our study who may or may not be immigrants, the existing immigration policies can indirectly influence the differing health culture of Hispanics and Caribbean-born Blacks where Caribbean-born Blacks may be less prone to access services because of perceived obstacles associated with the perception of an unfavorable socio-political climate.

Our finding that Bahamians had the worst odds for both outcomes against all referent groups was somewhat unanticipated, as they are considered to be less vulnerable compared to Haitians and other Caribbean groups residing in South Florida [19]. However, this may be attributed to a possible misclassification between Bahamians and Haitians. There is a relationship between Bahamas and Haiti and a history of Haitian immigration to the Bahamian islands [24,25,26]. Based on this historical precedent, it is possible that “Bahamian” cases may have been conflated with Haitian cases, or at minimum, a portion of the Bahamian cases may have Haitian heritage. Given the limitations of the dataset, this possibility could not be explored, but should be studied further as there may be differences in culture between the two countries that can influence health-seeking behavior and decision making.

Haitians living in rural areas were more likely to be retained in care and achieve viral suppression. There is evidence of rural areas having more social support [27] and less substance use [28], which are both factors that can improve persons living with HIV’s adherence to HIV medication and treatment [29]. Outside of Haitian immigrants, which were the only Caribbean immigrants with a population large enough to estimate rural/urban effects, it is difficult to postulate the effect of rural/urban status on non-retention and non-viral suppression among other Caribbean countries, as the percentages of non-Haitian Caribbean immigrants residing in rural areas in this population were low (average of 5% or less).

Trinidad and Tobago emerged as one of the countries with people living with HIV who were more likely not to be retained in care or not achieve viral suppression. Of note, the proportion of Trinidadians and Tobagonians with MSM as a mode of HIV transmission was higher than for any other Caribbean group (Table 1). The sample size of Trinidadian and Tobagonian immigrants living with HIV (n = 70) did not allow for within group analysis to assess the association between MSM mode of transmission/sexual behavior with the outcomes of interest, indicating another area that warrants further investigation.

Finally, although Jamaica has among the highest HIV rates in the Caribbean region [19], in this study, Jamaican immigrants living with HIV had the most favorable outcomes. It was beyond the scope of our analysis to determine what factors may have contributed to these results, but additional investigation into which factors assist Jamaican immigrants living with HIV in Florida to be retained in care and achieve viral suppression may help inform the development of efficacious interventions for immigrants living with HIV from other Caribbean countries.

Consistent with other HIV continuum care research, men did worse than women, and being diagnosed at an older age was a protective factor [30,31,32]. “Other” mode of HIV transmission and having an AIDS defining illness had significant associations with the two primary outcomes for all three countries, but the direction of association was different for these two variables. “Other” mode of transmission increased odds of non-retention and non-suppression, and having an AIDS defining illness was protective in this study. Previous research has found that individuals diagnosed with AIDS are more likely to be in care and be retained in care [33], but additional research is needed to determine the mechanism of association for AIDS diagnosis reducing risk of non-viral suppression. More information is also needed about “Other” mode of transmission (i.e., hemophilia, blood transfusion, and perinatal exposure), which may indicate other unknown underlying issues other than what is reported, including possible existing issues with the data capture instrument, or issues related to social stigma when people living with HIV are self-reporting this type of information [33,34]. Over 1300 cases were excluded from the analysis because of missing zip code which may suggest housing instability/insecurity that can also impact retention and viral suppression [35].

Although heterosexual mode of transmission accounted for most of the Caribbean cases, homophobia for MSM cases may impact engagement in HIV care. Regardless of sexual orientation, the need for privacy and anonymity because of fear of disclosure status in their communities, and associated stigma and discrimination can also deter routine engagement in care [36].

Factors driving non-retention and non-viral suppression are intersectional and complex for persons living with HIV who are also immigrants [37]. Primary data collection and qualitative methods on additional demographic factors and social determinants are needed to examine additional reasons for less retention and viral suppression in non-Hispanic Black Caribbean immigrants. Our analysis could not account for the role of acculturation, age of immigration, and migratory patterns of immigrants who may travel frequently to their countries of birth; nor could it account for second-generation born immigrants, or US-born Blacks who were ethnically non-Latino Caribbean immigrants. Additionally, our data may have had some differential selection for the retention analysis as cases who accessed services from ADAP and Ryan White programs may have had a higher probability of being selected for analysis. White Caribbean-born cases may have identified as non-Hispanic White; however, preliminary analysis indicated that over 95% of HIV cases from the Caribbean in this dataset were Black.

## 5. Conclusions

Florida continues to be one of the states most affected by HIV, having among the highest rates of new infections and people living with HIV, and Blacks having the highest HIV prevalence in the country [1]. This paper extends our previous findings about delayed diagnosis for Caribbean individuals living with HIV by exploring later stages of the HIV continuum of care and assessing non-retention and non-viral suppression disparity. The expanded analysis indicated that poorer health outcomes and lack of engagement in care for non-Hispanic Black Caribbean persons living with HIV may be related to factors other than race and be more related to cultural differences and socio-political context. HIV interventions that are developed to target this group should be comprehensive and take into consideration other social determinants that are exacerbating the disparity.

## Figures and Tables

**Table 1:** Demographic characteristics of persons living with HIV by race, ethnicity, and country of birth, retained in care, age 13 and older, Florida, 2000–2014, (n = 56,119).

Caribbean-Born Blacks						US Born Cases			
	Haiti	Jamaica	Bahamas	Trinidad & Tobago	Other ^a^	US Blacks	Hispanic	Non-Hispanic Whites	*p*-Value
	(n, %)	(n, %)	(n, %)	(n, %)	(n, %)	(n, %)	(n, %)	(n, %)	
Total [n (%)] ^b^	4491 (8)	701 (1.3)	265 (0.5)	70 (0.12)	122 (0.2)	22,362 (39.9)	13,859 (24.7)	14,249 (25.4)	<0.001
**Individual-level variables**									
Year of HIV diagnosis									
2000–2004	1852 (41.2)	216 (30.8)	122 (46.0)	30 (42.9)	52 (42.6)	8215 (36.7)	4641 (33.5)	5114 (35.9)	<0.001
2005–2009	1401 (32.2)	264 (37.7)	80 (30.2)	26 (37.1)	43 (35.2)	7532 (33.7)	4689 (33.8)	5001 (35.1)	
2010–2014	1238 (27.6)	221 (31.5)	63 (23.8)	14 (20.0)	27 (22.1)	6615 (29.6)	4529 (32.7)	4134 (29.0)	
Sex at birth									
Female	2225 (49.5)	289 (41.2)	141 (53.2)	27 (38.6)	47 (38.5)	9399 (42.0)	2466 (17.8)	2289 (16.1)	<0.001
Male	2266 (50.5)	412 (58.8)	124 (46.8)	43 (61.4)	75 (61.5)	12,963 (58.0)	11,393 (82.2)	11,960 (83.9)	
Age group at diagnosis									
13–24 years	306 (6.8)	78 (11.1)	42 (15.9)	5 (7.1)	9 (7.4)	5178 (23.2)	1775 (12.8)	1291 (9.1)	<0.001
25–49 years	3078 (68.5)	470 (67.1)	195 (73.6)	51 (72.9)	88 (72.1)	14,018 (62.7)	10,291 (74.3)	10,340 (72.6)	
≥50 years	1107 (24.7)	153 (21.8)	28 (10.6)	14 (20.0)	25 (20.5)	3166 (14.2)	1793 (12.9)	2618 (18.4)	
Mode of transmission									
IDU ^c^	78 (1.7)	26 (3.7)	11 (4.2)	0 (0)	9 (7.4)	1875 (8.4)	923 (6.7)	1401 (9.8)	<0.001
MSM	432 (9.6)	143 (20.4)	56 (21.1)	23 (32.9)	26 (21.3)	6883 (30.8)	8408 (60.7)	9747 (68.4)	
Heterosexual	3350 (74.6)	446 (63.6)	158 (59.6)	13 (18.6)	71 (58.2)	11772 (52.6)	3366 (24.3)	2250 (15.8)	
Other/unknown	631 (14.1)	86 (12.3)	40 (15.9)	34 (48.6)	16 (13.1)	1832 (8.2)	1162 (8.4)	851 (6.0)	
AIDS defining illness by 2015									
	2505 (55.8)	348 (49.6)	143 (54.0)	27 (38.6)	65 (53.3)	11137 (49.8)	5915 (42.7)	14,249 (25.4)	<0.001
**ZIP code tabulation area-level variables**									
Socioeconomic status (SES) index, quartiles									
1 (lowest SES)	2396 (55.3)	294 (43.8)	127 (49.6)	29 (43.9)	59 (50.4)	12,556 (58.1)	5094 (38.3)	3056 (22.2)	<0.001
2	1254 (29.0)	202 (30.1)	75 (29.3)	23 (34.9)	24 (20.5)	5342 (24.7)	3113 (23.4)	3664 (26.7)	
3	464 (10.7)	106 (15.8)	39 (15.2)	13 (19.7)	23 (19.7)	2448 (11.3)	3375 (25.4)	4061 (29.5)	
4 (highest SES)	216 (5.0)	70 (10.4)	15 (5.9)	1 (1.5)	11 (9.4)	1266 (5.9)	1720 (12.9)	2970 (21.6)	
Rural Urban Commuting Area classification									
Urban	4300 (95.8)	664 (94.7)	256 (96.6)	66 (94.3)	114 (93.4)	20,948 (93.7)	13,094 (94.5)	13,223 (92.8)	<0.001
Rural	191 (4.3)	37 (5.3)	9 (3.4)	4 (5.7)	8 (6.6)	1414 (6.3)	765 (5.5)	1026 (7.2)	

Note: ZCTA, ZIP code tabulation area; IDU, injection drug use; MSM, male to male sexual contact; RUCA, rural urban commuting area; SES, socioeconomic status. Percentage may not add up to 100 due to rounding; ^a^ Includes Guyana (n = 26), Turks & Caicos (n = 20), Barbados (n = 18), St. Lucia (n = 14), Dominica (n = 10), Antigua and Barbuda (n = 6), Grenada (n = 8), St. Vincent and the Grenadines (n = 7), Bermuda (n = 2), British Virgin Islands (n = 5), St. Kitts and Nevis (n = 4), Cayman Islands (n = 2); ^b^ Excludes cases diagnosed under 13 years of age (n = 270), missing month and year of HIV diagnosis (79), missing country of birth (n = 907), missing or invalid residential ZIP code (n = 1306), diagnosed in a correctional facility (n = 3195). ^c^ Includes cases reported as both IDU and MSM/IDU.

**Table 2:** Non-retention in care for Black Caribbean-born immigrants compared to US-born non-Hispanic Blacks, Hispanics, and non-Hispanic Whites in FL in 2015.

	US Born Blacks	Hispanics	Non-Hispanic Whites
	Referent Group	Referent Group	Referent Group
Country/region of birth	Adjusted OR (95% CI)	Adjusted OR (95% CI)	Adjusted OR (95% CI)
Bahamas	2.49 (1.91–3.23) **	2.69 (2.07–3.50) **	3.13 (2.40–4.10) **
Haiti	1.21 (1.12–1.30) **	1.31 (1.20–1.42) **	1.52 (1.40–1.66) **
Jamaica	0.89 (0.75–1.06)	0.97 (0.81–1.16)	1.13 (0.94–1.34)
Trinidad and Tobago	1.83 (1.10–3.04) **	1.98 (1.19–3.30) **	2.30 (1.38–3.83) **
Other Caribbean	1.52 (1.04–2.23)	1.65 (1.12–2.42) **	1.92 (1.31–2.81) **

Note: OR, odds ratio; CI, confidence interval; Adjusted Odds ratios: Controlling for individual-level variables (year of HIV diagnosis, sex at birth, age, race, mode of HIV transmission) and neighborhood-level variables (SES index and rural/urban status); ** *p* ≤ 0.001.

**Table 3:** Adjusted odds ratios and 95% confidence intervals for non-retention by selected characteristics, stratified by Caribbean country of birth, 2015.

	Bahamas	Haiti	Jamaica
	aOR, (95% CI)	aOR, (95% CI)	aOR, (95% CI)
**Individual-level variables**			
Year of HIV Diagnosis			
2000–2004	3.02 (1.42–6.44) **	1.90 (1.60–2.25) **	1.67 (1.05–2.65) **
2005–2009	2.14 (0.98–4.69) *	1.44 (1.20–1.72) **	1.60 (1.04–2.46) **
2010–2014	Referent	Referent	Referent
Sex at birth			
Male	1.10 (0.54–2.27)	1.46 (1.27–1.68) **	2.28 (1.50–3.48) **
Female	Referent	Referent	Referent
Age at HIV diagnosis			
25–49 years	1.07 (0.47–2.42)	0.91 (0.69–1.18) *	0.39 (0.23–0.68) **
≥50 years	0.49 (0.16–1.56)	0.87 (0.66–1.17)	0.25 (0.13–0.49) **
13–24 years	Referent	Referent	Referent
HIV transmission mode			
IDU ^a^	1.63 (0.38–6.98)	1.04 (0.63–1.72)	1.76 (0.71–4.37)
MSM	1.11 (0.46–2.64)	76 (0.60–0.97) **	79 (0.47–1.30)
Other/unknown	4.62 (1.78–12.18) **	3.46 (2.82–4.21) **	2.18 (1.28–3.71) **
Heterosexual	Referent	Referent	Referent
AIDS diagnosis			
No AIDS diagnosis by 2015	2.80 (1.49–5.25) **	3.24 (2.82–3.72) **	2.84 (1.95–4.12) **
**Neighborhood-level variables**			
Socio economic status			
1 (lowest SES)	0.80 (0.23–2.81)	.89 (0.65–1.23)	1.56 (0.83–2.92)
2	0.50 (0.14–1.84)	.85 (0.61–1.18)	1.42 (0.74–2.72)
3	0.54 (0.14–2.18)	1.05 (0.73–1.51)	1.67 (0.82–3.42)
4 (highest SES)	Referent	Referent	Referent
Rural vs. Urban			
Rural	-	0.21 (0.05–0.85) *	-

Note: IDU, injection drug use; MSM, male to male sexual contact; SES, socioeconomic status; aOR, adjusted odds ratio; CI, confidence interval; Odds ratios adjusted for individual-level variables (year of HIV diagnosis, sex at birth, age, race, and mode of HIV transmission), and neighborhood-level variables (SES index and rural/urban status); * *p* ≤ 0.05; ** *p* ≤ 0.001; ^a^ Includes cases reported as both IDU and MSM/IDU.

**Table 4:** Non- viral suppression for Black Caribbean-born immigrants compared to US-born non-Hispanic Blacks, Hispanics and non-Hispanic Whites in FL in 2015.

	US Blacks	Hispanics	Non-Hispanic Whites
	Referent Group	Referent Group	Referent Group
Country/region of birth	Adjusted OR (95% CI)	Adjusted OR (95% CI)	Adjusted OR (95% CI)
Bahamas	2.07 (1.59–2.69) **	2.70 (2.07–3.51) **	3.23 (2.48–4.21) **
Haiti	1.17 (1.09–1.26) **	1.52 (1.40–1.65) **	1.82 (1.68–1.98) **
Jamaica	0.83 (0.70–0.97) **	1.07 (0.91–1.27)	1.29 (1.09–1.52) **
Trinidad and Tobago	1.13 (0.68–1.86)	1.47 (0.89–2.42)	1.76 (1.06–2.90) **
Other Caribbean	1.30 (.89–1.89)	1.69 (1.16–2.46)	2.02 (1.39–2.95) **

Note: OR, odds ratio; CI, confidence interval; Adjusted Odds ratios: Controlling for individual-level variables (year of HIV diagnosis, sex at birth, age, race, mode of HIV transmission) and neighborhood-level variables (SES index and rural/urban status); ** *p* ≤ 0.001.

**Table 5:** Adjusted odds ratios and 95% confidence intervals for non-suppression by selected characteristics, stratified by Caribbean country of birth, 2015.

	Bahamas	Haiti	Jamaica
	aOR, (95% CI)	aOR, (95% CI)	aOR, (95% CI)
**Individual-level variables**			
Year of HIV Diagnosis			
2000–2004	2.26 (1.07–4.75) **	1.65 (1.40–1.93) **	1.40 (0.91–2.17)
2005–2009	2.13 (0.98–4.65)	1.42 (1.20–1.67) **	1.89 (1.26–2.84) **
2010–2014	Referent	Referent	Referent
Sex at birth			
Male	1.00 (0.48–2.06)	1.33 (1.16–1.52) *	2.37 (1.60–3.52) **
Female	Referent	Referent	Referent
Age at HIV diagnosis			
25–49 years	1.28 (0.56–2.88)	0.78 (0.60–1.00) *	0.46 (0.27–0.78) **
≥50 years	0.55 (0.17–1.73)	0.78 (0.59–1.03) *	0.29 (0.16–0.56) **
13–24 years	Referent	Referent	Referent
HIV transmission mode			
IDU	2.18 (0.48–9.93)	0.84 (0.52–1.36)	1.96 (0.79–4.84)
MSM	1.16 (0.48–2.76)	0.76 (0.61–0.96) *	0.69 (0.43–1.11)
Other/unknown	7.42 (2.25–22.44) **	2.51 (2.07–3.06) **	1.36 (0.81–2.84)
Heterosexual	Referent	Referent	Referent
AIDS Diagnosis			
No AIDS Diagnosis by 2015	2.59 (1.37–4.89) **	2.17 (1.91–2.48) **	1.96 (1.38–2.77) **
**Neighborhood-level variables**			
Socio economic status			
1 (lowest SES)	0.80 (0.23–2.78)	0.88 (0.65–1.20)	1.57 (0.87–2.84)
2	0.45 (0.12–1.63)	0.83 (0.61–1.14)	1.34 (0.73–2.44)
3	0.70 (0.18–2.78)	1.01 (0.72–1.44)	1.45 (0.74–2.83)
4 (highest SES)	Referent	Referent	Referent
Rural vs. Urban			
Rural	-	0.21 (0.06–0.78) *	-

Note: IDU, injection drug use; MSM, male to male sexual contact; SES, socioeconomic status; aOR, adjusted odds ratio; CI, confidence interval; Odds ratios adjusted for individual-level variables (year of HIV diagnosis, sex at birth, age, race, and mode of HIV transmission), and neighborhood-level variables (SES index and rural/urban status); * *p* ≤ 0.05; ** *p* ≤ 0.001.
